# Nickel Nanopillar Arrays Electrodeposited on Silicon Substrates Using Porous Alumina Templates

**DOI:** 10.3390/molecules25225377

**Published:** 2020-11-17

**Authors:** Matías Bejide, Patricio Contreras, Pia Homm, Boris Duran, José Antonio García-Merino, Andreas Rosenkranz, Juliano C. Denardin, Rodrigo del Río, Samuel A. Hevia

**Affiliations:** 1 Instituto de Física, Pontificia Universidad Católica de Chile, Casilla 306, Santiago 6904411, Chile; mabejide@uc.cl (M.B.); phomm@uc.cl (P.H.); jose.garcia@uc.cl (J.A.G.-M.); anrosenkranz@uc.cl (A.R.); 2 Centro de Investigación en Nanotecnología y Materiales Avanzados, Pontificia Universidad Católica de Chile, Casilla 306, Santiago 6904411, Chile; bgduran@uc.cl (B.D.); rdelrioq@uc.cl (R.d.R.); 3 Facultad de Química, Pontificia Universidad Católica de Chile, Casilla 306, Santiago 6904411, Chile; contreras@uc.cl; 4 Department of Physics, University of Santiago and CEDENNA, Santiago 9170124, Chile; juliano.casagrande@usach.cl

**Keywords:** nanomaterial, anodic aluminum oxide, well-ordered nanopores, electrochemical routes

## Abstract

Nickel nanopillar arrays were electrodeposited onto silicon substrates using porous alumina membranes as a template. The characterization of the samples was done by scanning electron microscopy, X-ray diffraction, and alternating force gradient magnetometry. Ni nanostructures were directly grown on Si by galvanostatic and potentiostatic electrodeposition techniques in three remarkable charge transfer configurations. Differences in the growth mechanisms of the nanopillars were observed, depending on the deposition method. A high correlation between the height of the nanopillars and the charge synthesis was observed irrespective of the electrochemical technique. The magnetization measurements demonstrated a main dependence with the height of the nanopillars. The synthesis of Ni nanosystems with a controllable aspect ratio provides an effective way to produce well-ordered networks for wide scientific applications.

## 1. Introduction

Complex nanostructures with well-organized matrix distribution and multi-modal physical behaviors are potential candidates for implementation in nanotechnology [1]. Specifically, nanowires (NWs) and nanopillars (NPillars) of Ni have many potential applications in a broad spectrum of areas, including data storage, magneto-electronics, quantum magneto-optics, biomedical science, spintronics, electrolysis, and lithium battery electrodes [2,3,4,5,6,7,8]. These technologies have a common interest in developing devices at low cost and with high throughput to produce diverse nanosystems [9]. In this context, electrochemical methods can be considered potential candidates for Npillars and NWs fabrication since they can be synthesized with various aspect ratios [10]. These structures were reported to enhance the magnetic properties with respect to Ni thin films because they present two principal axes of polarization [11]. In particular, Ni-NW and Ni-Npillars can be grown using well-distributed pores of anodic aluminum oxide (AAO) templates [12,13]. Generally speaking, Ni-NWs directly attached to semiconductors typically need several steps to be produced [14]. However, potentiostatic and galvanostatic electrochemical techniques can greatly simplify and increase the synthesis efficiency of Ni nanostructures [15].

Potentiostatic and galvanostatic electrochemical fabrication methods produce Ni-Npillars with magnetic properties due to the creation of polycrystalline grains with different shapes [16]. For example, Ni-NWs with diameters of 30 nm and 60 nm and a height of 10 µm have been electrodeposited on AAO templates using a potentiostatic procedure with AC and DC. The magnetic characteristics in the long axis of the wires, such as coercivity (||*H_c_*), increased with decreasing diameter [17]. Similar results are reported for the galvanostatic electrodeposition of Ni-NWs of 20 nm and 200 nm diameter and 60 µm height [18]. However, in these samples, the perpendicular saturation field (⊥*H_s_*) is smaller for the potentiostatic electrodeposited Ni-NWs compared to their galvanostatic counterpart [13]. Additionally, the magnetic properties of Ni-Npillars and NWs have an attractive implication due to the symmetrical angular propagation of electromagnetic waves [19]. This behavior has been well reported for Ni-NWs and can be explained by the strong correlation between the magnetic characteristics and shape anisotropy [20]. The effect of the morphology on the magnetic hysteresis loops is strongly pronounced for magnetically soft particles, such as Ni [21]. In addition, the preparation of Ni-Npillars with controlled dimensions covering areas on the order of cm^2^ attached to a semiconductor surface becomes an attractive topic in applied science [22]. For instance, Ni-Npillars have been used to develop potential lithium-ion battery electrodes [8,23]. Sn nanospheres were directly grown on Ni-Npillars to improve the volume charge in a lithium-ion battery anode; this alloy stabilizes the structure and suppresses the expansion during the reaction [24].

In this manuscript, a study of electrodeposited ordered arrays of Ni-Npillars on Si substrates using a porous alumina membrane (PAM) as a template is presented. The Ni-Npillars were directly grown onto the substrate, which is highly relevant from the scientific and technical points of view since it allows us to obtain cylindrical nanostructures with scalable ratios and immediate applications. The Ni-Npillars were fabricated by galvanostatic and potentiostatic electrochemical techniques and exhibited different growth mechanisms and magnetic properties, which depended on the respective fabrication method.

## 2. Results

**Figure 1:**
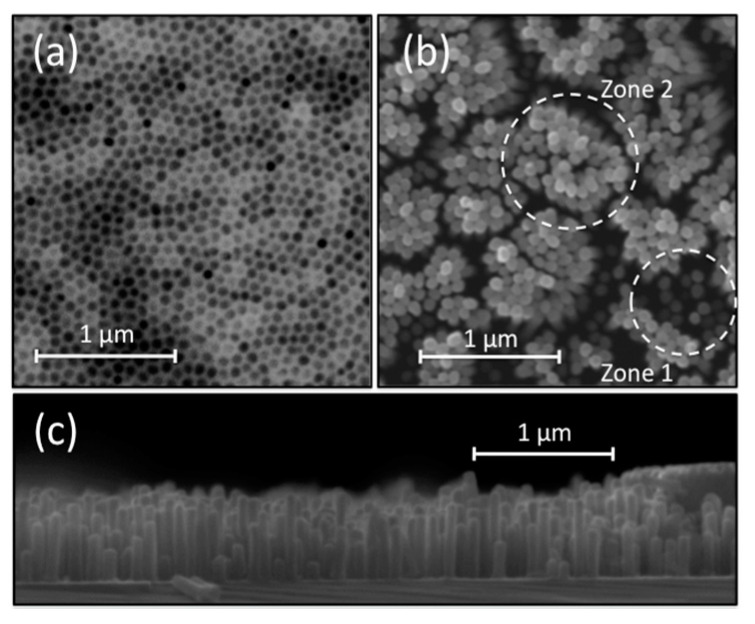
SEM micrographs. (**a**) Top view of the porous alumina membrane with Ni-Npillars electrodeposited inside the pores. (**b**) Top view and (**c**) side view of Ni-Npillars on Si substrate after removing the PAM template.

Figure 1 shows scanning electron microscopy (SEM) micrographs of the sample synthesized by galvanostatic electrodeposition at 1 C charge. Figure 1a shows a top view image of the PAM after Ni electrodeposition. A well-defined hexagonal pattern generated by the anodization process can be observed. Moreover, Figure 1b shows a top view of the Ni-Npillars after removing the PAM. The nanostructures follow the hexagonal arrangement of the template and show a quasi-circular cross-section with a mean diameter of 58.4 ± 8.8 nm and an interpore distance of 95.8 ± 7.2 nm, estimated based upon histograms of the AAO pattern distribution, as in Figure 1a. Initially, the Ni-Npillars tend to grow mainly parallel to each other, as observed in Zone 1 marked in Figure 1b. However, when their aspect ratio exceeds a critical value, the pillars tend to form bundles, as shown in Zone 2 of Figure 1b. Figure 1c shows a side view SEM micrograph of the Ni-Npillars after the template removal. As expected, the Ni-Npillars have grown perpendicular to the Si substrate with a rather broad height distribution. Only one sample was chosen to represent the morphological characteristics since similar trends were observed for all other samples, irrespective of the deposition technique and adjusted charge.

**Figure 2:**
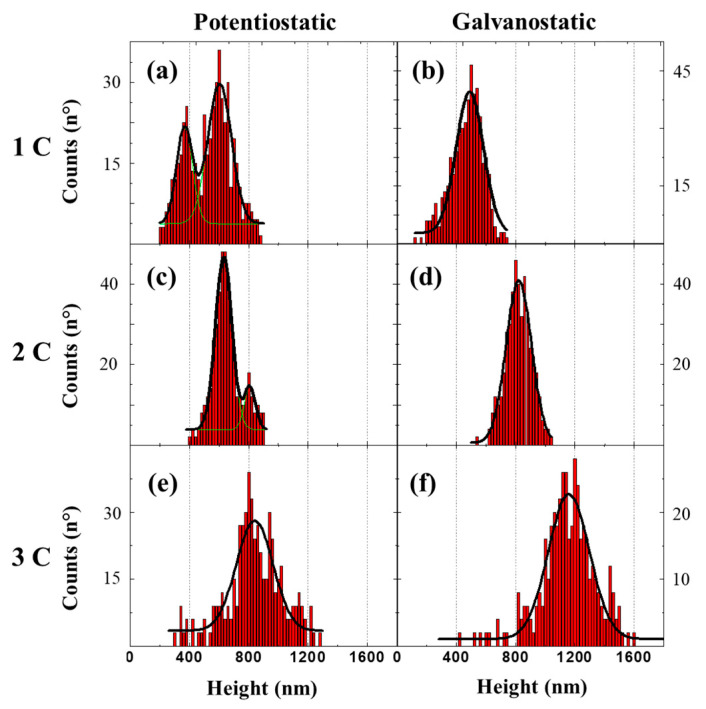
Histogram plots of the respective height distribution of all Ni-Npillars samples for both fabrication techniques as a function of the electrical charge. Potentiostatic at: (**a**) 1 C, (**c**) 2 C, and (**e**) 3 C. Galvanostatic at: (**b**) 1 C, (**d**) 2 C, and (**f**) 3 C.

Figure 2 summarizes the histograms of the height distribution of all Ni-Npillars samples as a function of the electrical charge and the electrodeposition method. This information has been obtained from a set of cross-section SEM micrographs, as shown in Figure 1c. The samples fabricated by the potentiostatic process (Figure 2a,c,e) demonstrate a rather broad height distribution, with the presence of a double peak for the two first synthesis charges. For the array with a synthesis charge of 2 C (Figure 2c), a much less pronounced double peak structure is seen, with one peak being much pronounced, having a mean height of 666 nm. In the case of the array with a synthesis charge of 3 C, a wider single peak distribution can be seen, with an average height of 844 nm (Figure 2e). For samples fabricated with the galvanostatic method, a synthesis charge of 1–3 C (Figure 2b,d,f), generally narrower single-peak distributions with mean values of 477 nm, 828 nm, and 1157 nm respectively, can be observed. For the galvanostatic depositions, the Ni-Npillars distribution tends to be wider, with an increase in the height mean value. In contrast, the potentiostatic technique generates high current densities with a high growth rate at the beginning of the process, leading to a large number of crystal nuclei [25].

**Figure 3:**
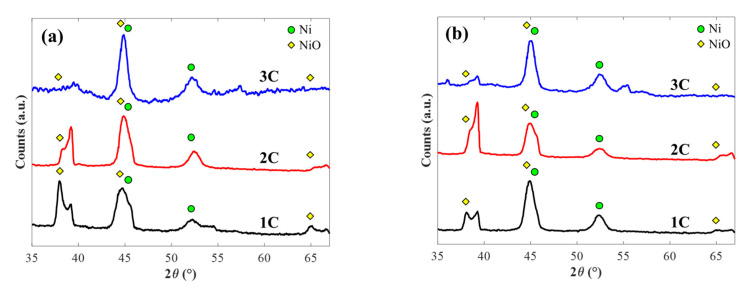
XRD measurements by grazing incidence for all samples as a function of the electrical charge for (**a**) potentiostatic and (**b**) galvanostatic electrodeposition.

Figure 3 depicts the resulting XRD measured by grazing incidence for all fabricated samples. At first glance, similar diffraction peaks can be found irrespective of the fabrication technique and electrical charge. Samples fabricated at 3 C of synthesis electrical charge, regardless of the used technique, demonstrate small differences in their spectra, which can be traced back to the formation of NiO during air exposure [26,27]. To analyze the crystallographic nature of the Ni nanostructures, the spectra of Figure 3 was deconvoluted. The double peaks observed near 38° and 65° correspond to a mixture of the same diffraction maxima, generated by two slightly different X-ray frequencies, CuKα and CuKβ. Moreover, the band close to 45° has two contributions, which can be attributed to Ni and NiO, thus resulting in a broader and asymmetric peak. For further analysis, all double and asymmetric peaks were deconvoluted, and only the bands belonging to CuKα radiation were selected. The exposed spectra exhibit five distinguished peaks: two for Ni (45.2° and 52.3°) and three for NiO (38.4°, 44.8°, and 64.8°). Similar values were reported elsewhere [28,29,30]. The respective maximum positions indicate the type of unit cell and lattice parameters for Ni and NiO. The unit cell of both materials was determined to be face-centered cubic (fcc) and polycrystalline, with an interatomic distance of 3.53 Å for Ni and 4.67 Å for NiO, calculated using Bragg’s law [31]. Moreover, by computing the full width at half maximum (FWHM), average values of 1.15 rad for potentiostatic and 1.07 rad for galvanostatic samples were estimated. Furthermore, using the Debye–Scherer equation [32], the calculated grain sizes of the Ni-Npillar arrays were 8.3 nm and 9.6 nm for the potentiostatic and the galvanostatic routes, respectively.

**Figure 4:**
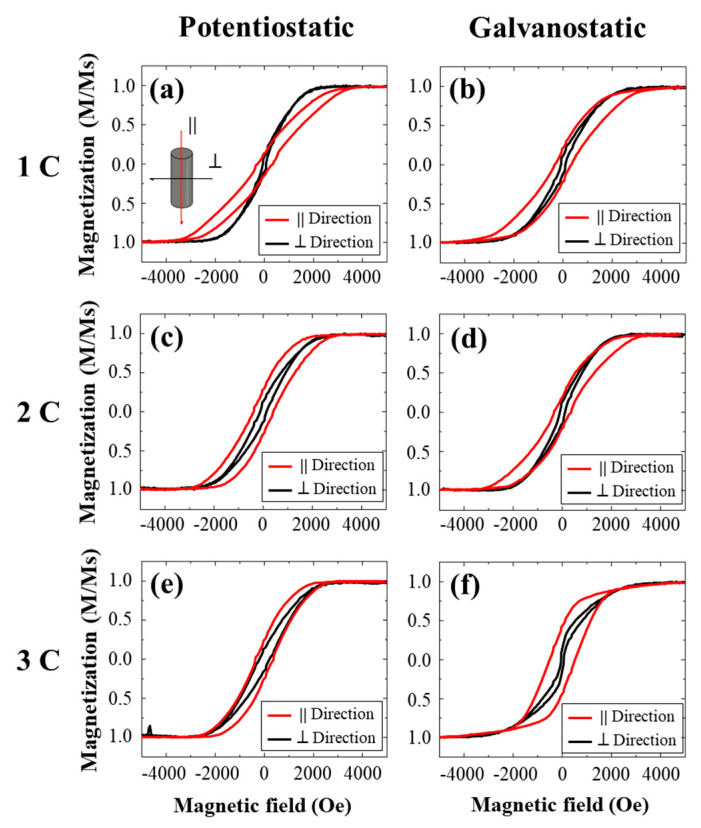
Hysteresis curves of the deposited Ni-Npillars with a parallel alignment external magnetic field (red) and perpendicular alignment (black). Potentiostatic at: (**a**) 1 C, (**c**) 2 C, and (**e**) 3 C. Galvanostatic at: (**b**) 1 C, (**d**) 2 C, and (**f**) 3 C.

Figure 4 shows a set of hysteresis curves measured for two different directions of the external magnetic field with respect to the long axis of the Npillars. The inset in Figure 4a shows the parallel (||) and perpendicular (⊥) room temperature magnetization isotherms. Based upon these curves, a change of the magnetic behavior can be observed depending on the fabrication technique and parameters. It is observed that the easy axis is perpendicular to the Ni-Npillars orientation for the 1 C potentiostatic sample and parallel to the pillars axis for the 2 C and 3 C samples. Moreover, there is no preferent magnetization to the wire axis for the 1 C and 2 C galvanostatic samples, but an easy axis parallel to the pillar axis is revealed for the 3 C galvanostatic sample. For both fabrication techniques, the saturating field decreases in the parallel direction as the synthesis charge increases from 1 C to 3 C. In general, for both axes, an increase in the charge promotes a bigger coercivity. In the same direction, from Figure 2 it is observed that the mean heights (*L*) of the Ni-Npillars increase with the charge level. This implies that the magnetic response can be tuned with the morphological aspect ratio of the nanostructures [33]. To study the interdependency between the parameters, such as the coercivity field (*H_c_*), saturation field (*H_s_*), remanence (*M*_r_), and the mean heights (*L*) of the Ni-Npillars, the correlations between these variables have been estimated. The respective summary is given in Table 1.

Based on Table 1, it is possible to observe that parallel magnetic properties are strongly related. These relations may be attributed to the well-correlated height of the Npillars. It is essential to distinguish that ||*H_s_* has a good correlation with all variables (negative absolute value), in contrast to ⊥*H_s_*, which cannot be related to other properties since every pillar is above a critical length (*L_c_*). It is expected for ⊥*H_s_* to be affected if the Npillars are below *L_c_* due to an in-plane magnetic anisotropy. This value can be estimated as *L_c_* = 1.27 *D*^3^/*d*^2^, where *D* is the interpore distance and *d* is the diameter pore [34]. The critical value from the average parameters of this work was computed at 283.2 nm, which is less than every Npillar height. Moreover, there is no good correlation between the height of the Npillars and ⊥*H_c_* [35]. Nevertheless, these Npillars are polycrystalline and do not show an extraordinary preferential magnetic orientation [36].

Furthermore, since the height of the Ni-Npillars determines almost every magnetic parameter, similar loop behaviors can be expected for both directions for the 1 C potentiostatic and 1 C galvanostatic, as well as for the 3 C potentiostatic and 2 C galvanostatic samples. From Figure 4a,b, a similar loop for the parallel direction can be observed. However, the potentiostatic array shows a smaller saturation field in the perpendicular direction. Figure 4d,e present different behaviors, despite their similar average height. Moreover, the potentiostatic samples exhibit a more sensitive dependence in the perpendicular direction and a monotonic increase in the ||*H_c_*, with respect to the synthesis charge. This can be attributed to different behaviors induced by distinct polycrystalline grain growth in the Ni-Npillars, depending on the deposition method. This phenomenon can explain the observed discrepancies. For instance, if the potentiostatic samples grow with more elongated grains in the perpendicular direction, the ⊥*H_c_* is expected to increase due to more overlapping grains or increased heights [22]. Additionally, the easy axis orientation perpendicular to the applied field for the 1 C sample could be explained with ellipsoid-like grains, which exhibit anisotropy in their long axis [33,36,37]. In contrast, the galvanostatic samples with 1 C and 2 C are more isotropic, while the sample with 3 C presents the highest Npillars, thus implying the greatest anisotropy. The effect in the 3 C sample can be mainly attributed to the large height that makes it easy to magnetize in the wire axis. Moreover, the rather similar behavior observed in Figure 4b,d, although showing a height difference of 331 nm between the individual nanostructures, can be associated with a more spherical grain shape distribution for the galvanostatic sample [33]. In this context, it is also important to point out that potentiostatic and galvanostatic methods tend to generate different crystalline phases [16]. Potentiostatic routes have been shown to generate Ni-NWs with ellipsoidal grain shapes exhibiting similar hysteresis loops in perpendicular and parallel directions [19,22]. In contrast, the galvanostatic electrodeposition of Ni and Ni-W promotes multilayer deposition with a multi-nuclear growth mechanism, thus generating more symmetric grains [38,39]. Additionally, no previous reports have been found for which Ni-NWs or Ni-Npillars were electrodeposited by these methods, which could help to support our claims regarding the different grain shapes. Therefore, further morphological characterization, such as a transmission electron microscopy study, is required to determine the Ni and NiO phases, as well as the texture of the grains.

Finally, it is worth mentioning that other Ni nanostructures have been used to coat semiconductors, leading to interesting magnetic effects. For instance, carbon nanotubes decorated with Ni nanoparticles have been applied for sensing quantum wave propagations in a variable magnetic field [40]. The advantage of Ni against carbon allotropes is their adhesive strength on substrates. These values have been reported around 3.5 GPa for Ni-NWs [41] and 714.1 MPa for nanotubes [42]. Therefore, the development of scalable Ni-Npillars seems to be an interesting way to design magnetic networks, which are good candidates for developing magnetic memories, magneto-optical switches, and thermo-optical amplifiers [43,44]. Moreover, considering that Ni-Npillars have a high surface area and tunable aspect ratio, they can be a useful platform for the growth of NiO on the walls of the pillars to obtain multifunctional metal/oxide devices. This is feasible to undergo, even at room temperature, and was observed in the XRD-spectra of Figure 3. The size of the oxide grain can be controlled in polycrystalline Ni and is well described by the Mott-Cabrera model for low-temperature oxidation [45]. The oxidation kinetics on a polycrystalline Ni surface occurs by ion transport on the metal phase and mostly happens at the grain boundaries [45]. Since this process is viable in Ni-Npillars, they are good candidates to support NiO as a nanostructured base for lithium-ion battery anodes [46,47]. The efficiency of the ion exchange is highly affected by the grain size of the NiO and is crucial to developing these electrodes [48].

## 3. Materials and Methods

### 3.1. Fabrication of Porous Alumina Membranes onto Si

To fabricate PAMs, high purity Al (99.995%) was deposited on polished *n*-type Si (100) wafers with 1–10 Ω/cm by electron beam evaporation under high vacuum conditions (~10^−7^ Torr base pressure). Afterward, the Al film with a thickness of 3 μm was subjected to a double anodization process, leading to a well-order porous alumina layer, as described in [49]. The pore characteristics can be tuned by adjusting the anodization time and the chemical etching procedure. A pore pattern with a 60 nm diameter was created using an etching process with 5 wt% of phosphoric acid solution at room temperature for 60 min. This step removes the oxide layer at the bottom of the PAM pores, leaving the Si exposed for the Ni nucleation.

### 3.2. Synthesis of Ni Nanopillars

To study the growth mechanism, Ni-Npillars were synthesized by potentiostatic and galvanostatic electrochemical techniques using the same cell configuration. The deposition procedures were carried out using an electronic analyzer CHI1140B provided by CH Instruments (Texas, USA) with a working solution of NiSO_4_·6H_2_O (0.050 M, Sigma-Aldrich, MO, USA), H_3_BO_3_ (0.040 M, Merck, MO, USA), and Saccharin (0.016 M, Sigma-Aldrich), purged with Ar for 10 min. The Si substrates, previously prepared with the PAM mask, were used as a working electrode with an exposed area of 0.4 cm^2^. Additionally, a stainless steel plate was used as a counter electrode and an Ag/AgCl electrode as a reference. Galvanostatic and potentiostatic electrodepositions were carried out using a current density of 1.875 mA/cm^2^ and a potential of −1.75 V, respectively. Both methods were employed until charges of 1 C, 2 C, and 3 C were reached. After the Ni nanostructures were deposited, the PAM templates were removed with a solution of NaOH 1.25 M for 3 h. Finally, the samples were cleaned with MiliQ water to remove any trace of oxide remaining on the Npillars.

### 3.3. Characterizations

The samples were characterized by scanning electron microscopy (SEM) using a LEO model VP1400 (LEO Electron Microscopy Inc., New York, USA). The atomic structure of the Ni-Npillars was performed by X-ray diffraction (XRD), using a diffractometer D8 Advance provided by Bruker (Massachusetts, USA) and equipped with CuKα radiation source (1.56 Å). Additionally, a custom-built alternating force gradient magnetometer (AGM) was used at room temperature to study the anisotropic magnetic properties. Two distinguished propagation directions of the magnetic field were considered: parallel (||) and perpendicular (⊥) to the long axis of the Npillars.

## 4. Conclusions

Within this work, galvanostatic and potentiostatic techniques have been used to successfully grow Ni-Npillars onto Si substrate using a PAM as a template. The periodic pore distribution of the AAO is crucial to obtain Npillars with a high aspect ratio to create an efficient magnetic nanosystem. The electrodeposition technique and the charge level define the mean height distribution of the Ni-Npillars. The XRD confirmed that the grain size of the polycrystalline structures is bigger in the galvanostatic samples. The magnetic properties show a strong dependence on the growing electrochemical method and the mean height of all samples. Npillar arrays with similar heights but fabricated by different electrodeposited techniques exhibit different magnetization hysteresis in the perpendicular direction. Differences related to the electrodeposition method led to the formation of unique Ni nanostructures with remarkable vectorial magnetization dependence, which can be considered for the development of multimode systems.

## Figures and Tables

**Table 1:** Parallel and perpendicular magnetic properties of Ni-Npillars and their correlation variables. The blanks are either one between the same variable, or the same value with respect to its symmetry.

Method	Potentiostat			Galvanostat			Correlation					
	1 C	2 C	3 C	1 C	2 C	3 C	*L*	||*H_c_*	⊥*H_c_*	||*M*_r_	⊥*M*_r_	||*H_s_*
*L* (nm)	486	655	818	481	812	1130	-	-	-	-	-	-
||*H_c_* (Oe)	633	814	741	703	690	1036	0.81	-	-	-	-	-
⊥*H_c_* (Oe)	133	263	427	187	212	288	0.5	0.39	-	-	-	-
||*M*_r_ (M/Ms)	0.13	0.28	0.25	0.21	0.18	0.43	0.81	0.99	0.48	-	-	-
⊥ *M*_r_ (M/Ms)	0.11	0.13	0.14	0.12	0.13	0.15	0.92	0.79	0.75	0.83	-	-
||*H_s_* (Oe)	3641	3000	2738	3391	3296	2440	−0.87	−0.87	−0.79	−0.91	−0.94	-
⊥*H_s_* (Oe)	2380	2293	2738	2811	2640	2851	0.49	0.37	0.34	0.41	0.49	−0.45
